# Accounting for failure: risk-based regulation and the problems of ensuring healthcare quality in the NHS

**DOI:** 10.1080/13698575.2016.1192585

**Published:** 2016-06-27

**Authors:** Anne-Laure Beaussier, David Demeritt, Alex Griffiths, Henry Rothstein

**Affiliations:** ^a^ Department of Geography, King’s College London, London, UK; ^b^ School of Management and Business, King’s College London, London, UK

**Keywords:** risk, regulation and governance, healthcare quality, National Health Service (NHS), Care Quality Commission

## Abstract

In this paper, we examine why risk-based policy instruments have failed to improve the proportionality, effectiveness, and legitimacy of healthcare quality regulation in the National Health Service (NHS) in England. Rather than trying to prevent all possible harms, risk-based approaches promise to rationalise and manage the inevitable limits of what regulation can hope to achieve by focusing regulatory standard-setting and enforcement activity on the highest priority risks, as determined through formal assessments of their probability and consequences. As such, risk-based approaches have been enthusiastically adopted by healthcare quality regulators over the last decade. However, by drawing on historical policy analysis and in-depth interviews with 15 high-level UK informants in 2013–2015, we identify a series of practical problems in using risk-based policy instruments for defining, assessing, and ensuring compliance with healthcare quality standards. Based on our analysis, we go on to consider why, despite a succession of failures, healthcare regulators remain committed to developing and using risk-based approaches. We conclude by identifying several preconditions for successful risk-based regulation: goals must be clear and trade-offs between them amenable to agreement; regulators must be able to reliably assess the probability and consequences of adverse outcomes; regulators must have a range of enforcement tools that can be deployed in proportion to risk; and there must be political tolerance for adverse outcomes.

## Introduction

Over the last 30 years, the desire to improve the quality of healthcare has been the one constant amidst near ceaseless reforms to the British National Health Service (NHS). Whereas Thatcher-era reforms of the 1980s looked to dedicated managers and to the discipline of the internal market ‘to secure the best deal for patients and the community health within available resources’ (Griffiths, 1983, para. 3), subsequent governments created independent watchdogs, charging a succession of steadily more powerful regulatory agencies with monitoring quality and ensuring standards. But the experience of healthcare regulators has not been a happy one. External regulatory inspection and oversight have proved both costly and controversial, with critics highlighting the perverse consequences for care quality of ham-fisted regulation (Bevan & Hood, 2006). In response to such difficulties, healthcare regulators have increasingly adopted so-called ‘risk-based’ approaches to regulation. In this article, we examine how and why these risk-based approaches have failed to improve the regulation of healthcare quality.

The central conceit of risk-based regulation is that regulators cannot, and indeed should not even try, to prevent all possible harms. Instead, regulatory interventions should focus on controlling the greatest potential threats to achieving regulatory objectives, as determined through ex ante assessments of their probability and consequences. Prioritising regulatory activities in this risk-based way promises to make regulation more effective and proportionate (Rothstein, 2006). To that end, successive UK Governments have enthusiastically promoted risk as a central principle of ‘better regulation’ and since 2008 have mandated its use for targeting regulatory inspection and enforcement activities (BIS, 2014). Accordingly, risk-based approaches now feature prominently in the regulation of many aspects of healthcare, from certifying medical professionals and assuring the safety of devices to managing financial failures by NHS Trusts (Challoner & Vodra, 2011; Lloyd-Bostock & Hutter, 2008; Murrary, Imison, & Jabbal, 2014; Phipps, Noyce, Walshe, Parker, & Ashcroft, 2011).

Care quality regulation is perhaps the most notable example of this trend. Building on the statistical surveillance techniques developed by its predecessors, the Care Quality Commission (CQC) has sought to make its regulatory interventions more ‘risk-based’ by targeting those providers at greatest statistical risk of failing to meet the required standards. Despite the technical sophistication of these risk-based prioritisation systems, however, the CQC and its predecessors were severely criticised for failing to prevent several high-profile breakdowns in care quality – most notably at Mid-Staffordshire Trust (Francis, 2013) – prompting two successive Chairs and a Chief Executive of the CQC to resign between its creation in 2008 and 2012. In response, the CQC fundamentally reformed its regulatory approach and redoubled its efforts to be risk based (Care Quality Commission (CQC), 2013b). But its latest system for prioritising hospital inspections was found not to work (Griffiths, Beaussier, Demeritt, & Rothstein, 2016), and the CQC (2015e) is currently revising its risk-based model once again. In this article, we examine the reasons for these failures in order to improve understanding of the conditions necessary for successful risk-based regulation.

## The promise and problems of risk-based regulation

Before examining why risk-based regulation has not worked for healthcare quality, it is worth reviewing what it promises to achieve. Promoted internationally by organisations, such as the US Office of Management and Budget (OMB), the World Trade Organization (WTO), the Organisation for Economic Cooperation and Development (OECD), and the European Union (EU), risk-based approaches have become a common trope of regulatory reform programmes around the world; marking a shift in the relationship between risk and regulation (Rothstein, Borraz, & Huber, 2013). Risk is no longer simply the *object* of regulatory control, be it potential harms such as pharmacological side effects and workplace accidents or from newly defined threats such as radicalisation or invasions of digital privacy. Rather, risk has also become a central *means* for making regulation socially optimal by using formal risk assessments of probability and consequence both to define regulatory objectives as well as target only the greatest threats to achieving those objectives (Black & Baldwin, 2010; OECD, 2010). To do otherwise – so the argument goes – can be grossly inefficient, inadvertently create other risks, or distract attention from more serious problems. By redefining what adverse outcomes can be counted as successes or failures through the language of acceptable or unacceptable risk, risk-based approaches also provide a means for regulators to account for the limits of what regulation can, and should, seek to achieve (Dodds & Kodate, 2011; Power, 2007; Rothstein, Huber, & Gaskell, 2006).

To that end, risk-based ideas and instruments can be used to reorganise the three essential feedback control functions that, from a conventional cybernetics perspective, define a regulatory regime (Hood, Rothstein, & Baldwin, 2001). First in *setting regulatory goals*, risk-based principles and instruments can be used to define qualified – rather than absolute – regulatory standards. One example is the UK’s long-standing workplace safety requirement that workers should be protected against harm only ‘so far as is reasonably practicable’; a legal principle that explicitly balances the probability and consequence of harms occurring against the cost, time, and effort of reducing potential harms further (Demeritt, Rothstein, Beaussier, & Howard, 2015). Second, risk instruments can be used to organise *information gathering* about the extent to which a regulated system is meeting regulatory goals. Examples include probabilistic sampling to scrutinise financial transactions that are most likely to be in breach of money laundering regulations (Wesseling, 2013) or probabilistically qualifying predictions of whether and where it will flood (Demeritt, Nobert, Cloke, & Pappenberger, 2013; Kuklicke & Demeritt, 2016). Finally, risk-based approaches to *enforcement and implementation* use risk to calibrate a ‘pyramid’ of escalating regulatory interventions in proportion to the probability and consequence of non-compliance with regulatory goals (Ayres & Braithwaite, 1992). Thus, the ‘pupil premium’ in England targets additional resources to schools with more economically disadvantaged pupils ‘at risk’ of failing (National Audit Office (NAO), 2015b), while actuarial sentencing uses formal risk assessments of offender recidivism to determine the nature and length of custodial sentences handed down to convicted criminals (Hannah-Moffat, 2013).

However, research on risk-based regulation in other domains highlights a number of potential problems in applying this approach to regulating healthcare quality. First, there is the question of ‘risks to what?’ In animal welfare regulation, for example, goal ambiguity has made it difficult both to define clear standards and to calibrate regulatory interventions in proportion to risk of non-compliance (Escobar & Demeritt, 2016). In healthcare, there are similar ambiguities about the very meaning of quality and thus how a ‘risk’ to it might be defined. Although some medical experts insist ‘quality can be defined and measured’ objectively (Chassin & Galvin, 1998, p. 1001), in practice conceptions of quality have rarely attracted consensus among the policy and medical communities or wider publics. The ambiguity is captured by Donabedian’s (1988) influential triadic model, which frames healthcare quality through three distinct dimensions of ‘structures’ (including facilities, staffing, budgets, and capacities), ‘processes’ (for example, care procedures, systems, and organisations), and ‘outcomes’ (such as clinical effectiveness, public health, and satisfaction). Indeed, quality regimes in other domains, such as higher education, have experienced similar difficulties in defining their objects and thus in organising quality regulation according to risk (Gibbs, 2010; Griffiths, 2016; Huber & Rothstein, 2013).

Second, risk-based regulation is also difficult without agreement on the acceptability of potential adverse outcomes. In the food safety domain, for example, it has been much harder for the various interest groups to agree on acceptable risk thresholds than in workplace health and safety, where there are just two principle interest groups – employers and employees – who share a common interest in agreeing regulatory trade-offs between cost and safety in order to protect profits and job security (Demeritt et al., 2015). Arguably, healthcare is like food safety: it has high public salience and involves numerous organised interest groups with little incentive to compromise their preferences. As Sir Donald Irvine (2006) – a former President of the General Medical Council (GMC) has commented:

The [risk-based] strategy is not compatible with the concept of a guarantee to the public of a good doctor for all … The government will need to demonstrate that it has the public’s fully informed consent if it decides to support this line. After all it is patients, not doctors, who may be killed or injured by poor doctoring. (p. 431)

Even when it is possible to agree about the ex ante acceptability of potential adverse outcomes, those regulatory compromises are sometimes forgotten when adverse outcomes actually occur. For example, during the extensively televised 2014 flooding of the Somerset Levels in South West England, the prime minister disavowed the Environment Agency’s risk-based strategy for prioritising national flood defence investments in proportion to risk, insisting that ‘money is no object’ and allocating an additional £20 million for dredging and other local alleviation schemes in Somerset that failed the Agency’s cost–benefit test (Demeritt, 2014). The National Institute for Health and Care Excellence has faced similar difficulties upholding its recommendations about which drugs should be available on the NHS in the face of political pressure from patient groups keen to have life-saving cancer drugs funded regardless of their cost-effectiveness (Ferner & McDowell, 2006).

Third, methodological challenges in assessing risk can make risk-based regulation difficult to implement. In the domain of water quality regulation, for example, risk-based strategies have struggled to deal with non-point water pollution, because the huge number and variety of potential sources make it difficult to identify and effectively target which sources pose the greatest risk (Black & Baldwin, 2012). Risk-based targeting is particularly difficult when the underlying base rate of occurrence for a hazard is very low. Thus, even if psychiatric risk assessments were much more sensitive than they actually are, the rate of violent psychosis is so low that the number of people falsely identified as posing a risk to others would still be many times larger than the number correctly identified as dangerous and involuntarily committed as a result (Szmukler & Rose, 2013). Just like medical diagnosis (Swets, 1988), regulation is similarly plagued by difficult trade-offs between type 1 (false positive) and type 2 (false negative) detection errors (Black, 2010). But even with the best data in the world, there is an irreducibly normative element to indicator construction, selection, and application, which makes them politically contentious. For example, in education (Acquah, 2013), the Conservative-led government scrapped the ‘contextual value added’ measure introduced in 2006 under Labour to take student affluence into account in school league tables on the grounds that it is ‘morally wrong to have an attainment measure which entrenches low aspirations for children because of their background’ (Department for Education, 2010, para. 6.13) .

Finally, there can also be legal and institutional obstacles to organising regulation in proportion to risk. For example, the European Court of Justice recently banned insurers from charging differential premiums to male and female drivers based on actuarial differences in accident rates between the sexes (Edmonds, 2013). Similarly anti-racist campaigners have long complained that risk-based profiling of potential terrorism suspects (Amoore, 2006), like stop-and-search policing more generally (Bowling & Phillips, 2007), is racially discriminatory and thus illegal under equalities legislation. Even when it is legally permissible, calibrating institutional responses in proportion to risk can conflict with the wider matrix of political and organisational pressures. Thus, government demands that universities vet external speakers to reduce the risk of Islamic radicalisation have met with fierce resistance from champions of academic freedom and free speech (Times Higher Education, 2016).

In the analysis that follows, we will consider whether and how these difficulties experienced in other domains of risk-based regulation manifest themselves in the particular case of healthcare quality in England. In particular, we will examine the use of risk-based policy instruments for defining, assessing, and enforcing regulatory standards and explain why they have failed to achieve the wider goals of healthcare quality regulation.

## Methods

The paper adopts a qualitative methodology combining policy document analysis and in-depth interviews in 2014–2015 with 15 high-level informants closely involved in the design, implementation and reform of successive care quality regulators. Those informants include past and serving senior civil servants, regulators, NHS officials, politicians, and professional group representatives. Interviews were conducted following a protocol approved by the King’s College London Research Ethics Committee (REP(GSSHM)/13/14-5). Interviews were recorded, transcribed, and thematically coded for comparison against documentary sources. Using both source and method triangulation to ensure validity (Baxter & Eyles, 1997), we draw on this dataset to systematically analyse the technical and contextual challenges to applying risk-based concepts and policy instruments to the three functional components of healthcare quality regimes: *defining* the goals of quality regulation; *assessing* quality; and *enforcing* compliance with those quality goals. In the following section, we start by setting out the background to healthcare quality regulation and then go on to examine each regulatory component in turn.

## Findings: healthcare quality regulation in the NHS

### Background and history

Care quality has only been formally regulated since the turn of the millennium. For most of its history, care quality in the NHS was left to doctors, overseen only by the centuries-old royal medical colleges. Responding to increasing public complaints and the scandal over high death rates among infant heart patients at Bristol Royal Infirmary (Alaszewski, 2002; Bevan, 2008), the 1999 Health Act imposed a new statutory ‘Duty of Quality’ on NHS Trust Boards. This duty was enforced through the creation of a new independent regulatory regime, which has seen a remarkable churn of regulators, mandates, standards, and practices.

The first quality regulator was the Commission for Health Improvement (CHI). Its mandate was to advise on and review the ‘clinical governance’ processes put in place by NHS Trusts to fulfil their Duty of Quality. Although CHI had no formal sanctioning powers, it publicly star-rated NHS Trusts based on their performance against key national targets (Bevan & Cornwell, 2006, p. 10) and its rolling peer reviews identified serious quality assurance failures, prompting a number of hospital chief executives to resign (Nuffield Trust, 2013).

The 2003 Health and Social Care Act replaced CHI with a more powerful regulator, the Healthcare Commission (HCC), as part of wider NHS reforms aimed at increasing capacity through competition and the involvement of private healthcare providers. To that end, the new regulator was given enhanced enforcement powers and a dedicated professional inspectorate to publicly rate quality and compliance with a set of new detailed standards, performance targets, and quality assurance requirements as part of its ‘Annual Health Check’ of NHS Trusts. Faced with overseeing a large number of complex healthcare organisations, the HCC commissioned the first risk-based monitoring system for detecting statistical abnormalities in NHS hospital performance and triggering unannounced inspections (Bevan & Cornwell, 2006).

The HCC was in turn abolished by the 2008 Health and Social Care Act, which consolidated quality regulators from across the health, social, and mental healthcare sectors under a new Care Quality Commission (CQC). Whereas CHI had been responsible for monitoring care quality in just 500 or so NHS organisations, the CQC was responsible for licencing and assuring care quality outcomes in almost 50,000 registered health and social care providers across England. In developing ‘a generic regulatory model that could be applied to all sectors’, the CQC (2012) adopted a number of explicitly risk-based strategies for organising its vastly expanded duties. Regular on-site inspections of all providers were abandoned in favour of risk-based prioritisation, as the number of compliance checks completed by its new generalist inspectorate ‘fell significantly’ (National Audit Office (NAO), 2011, p. 8). Targeting was underpinned by an expanded statistical surveillance system rating all providers for their risk of non-compliance with 16 separate standards.

Blistering criticisms of care quality regulation, however, not least from the Francis Inquiry (2013) into serious care failures at Mid-Staffordshire NHS Trust, led to new quality regulations and wholesale reforms of the CQC’s regulatory model. For its ‘new start’, the CQC (2013a) developed five new ‘tests’ of care quality, revised its statistical surveillance system, and returned to regular inspections conducted by new specialist inspectorates for hospitals, social care, and primary care. However, the new regulatory model has already run into trouble. In 2015, general practitioners (GPs) complained about how they were publicly risk rated (Millett, 2015) and then, in 2016, the first peer-reviewed evaluation of the CQC’s new surveillance system showed that it could not detect risks to care quality in hospitals (Griffiths et al., 2016).

In the following sections, we examine the historic problems facing healthcare regulators in using risk ideas and policy instruments to help define, assess, and enforce compliance with quality standards.

### Defining risk-based quality standards

The first set of problems facing risk-based approaches to regulating healthcare quality is in defining their goals in terms of acceptable risk. One challenge is that the concept of ‘quality’ is ambiguous and contested. Over the last 20 years, the Department of Health (DoH) has redefined statutory care quality frameworks four times, layering new definitions on top of old ones in response both to specific crises, and to changing government priorities about the trade-offs between treatment volume, cost containment, clinical outcomes, and patient satisfaction. Thus, the 1999 Health Act imposed the Duty of Quality on NHS Trusts in response to concerns that the DoH’s long-standing regime of productivity targets was compromising patient safety. Drawing on fashionable ideas of corporate governance, the Act required trusts to implement clinical governance controls ‘for the purpose of monitoring and improving the quality of health care which it provides to individuals’ (s.18.1). CHI’s clinical governance reviews consequently entailed checking the internal quality assurance processes of NHS Trusts against national standards set by professional bodies. Rather than replacing productivity targets, however, CHI’s process-centred approach to healthcare quality sat alongside them. Indeed, the DoH’s national target regime was given new emphasis by the publication of Ofsted-style ‘star-ratings’ for individual NHS Trusts, based upon their performance against key national targets, such as waiting times.

This dual quality regime, however, was criticised for emphasising processes and inputs over ‘what matters most to patients, carers and the public’ (DoH, 2005, p. 11). So in 2004, when the HCC was created, the DOH (2006, para. 16) issued a completely new set of 44 quality standards ‘described in terms of outcomes’, adding yet another layer to the quality ‘cake’. Reflecting the classical business management nostrum, ‘quality is what the customer says it is’, these new quality standards incorporated an explicit ‘patient focus’, including specific requirements on the quality of catering services and for ‘continuously improv[ing] the patient experience, based on the feedback of patients, carers and relatives’ (DoH, (2006), p. 32). At the same time, the DOH (2004, p. 9) also cut the number of performance targets NHS Trusts were required to meet from 62 to 20, and reduced their emphasis on inputs so that more than half focused on ‘health outcomes and patient experience’.

When the CQC assumed responsibility for regulating social care as well as healthcare in 2009, the government had to redefine quality regulations once again. The 44 quality standards used by HCC were boiled off into ‘a single coherent set’ of 16 more generic ones that could be applied across both the health and social care sectors (DoH, 2009, para. 1.4). However, those 16 essential quality standards lasted only a few years. After the Francis Inquiry (Francis, 2013, para. 1.130) called for ‘fundamental standards’ to be ‘set out in a clear manner so they can be understood and accepted by providers, patients and the public’, the government revised quality regulations again. A new statutory Duty of Candour on providers was introduced in 2014 and the 16 ‘essential quality’ standards were reduced to 11 ‘fundamental standards’, which the CQC currently assesses by asking if care is ‘Safe’, ‘Effective’, ‘Caring’, ‘Responsive’, and ‘Well-led’.

This instability of regulatory goals and quality definitions has made it difficult ‘to have a regulatory framework with integrity’, as one regulatory official explained to us. Each successive reform, he noted, ‘takes apart how regulation has been carried out’ and forces ‘us to go about designing these regulatory models basically from scratch’. In that context, it has been difficult to operationalise effective risk-based (or indeed any other) approaches to regulating what has essentially been a moving target.

A second challenge to defining the goals of regulation in terms of acceptable risk has been determining the boundary between acceptable and unacceptable outcomes. For example, regulators and the NHS have struggled to agree ex ante on how far it is reasonable to go to eliminate what are called ‘never events’, such as wrong site surgery, which occur with alarming regularity. In 2009, the first year in which they were systematically recorded, the CQC reported that there were 111 ‘never events’ across the England (CQC, 2014b). Although formally unacceptable, ‘never events’ are in practice tolerated because the measures required to eliminate them, such as abandoning surgical interventions altogether, are even less acceptable than the risk of never events occurring.

With their emphasis on qualified, rather than absolute regulatory goals, risk-based approaches promise to help manage such trade-offs by using *ex ante* assessments of probability and consequence to determine the acceptability of different potential quality outcomes. However, the politics of healthcare have not been favourable to agreeing trade-offs between healthcare’s ‘iron triangle’ of access, quality, and cost (Kissick, 1994). As a former junior health minister explained to us, defining acceptable risks to healthcare quality ‘is a big challenge … [it is] … very hard for a regulator to come out and say well, I’m sorry, but we can’t guarantee your safety’. The CQC’s current standards, *safe, effective, caring, responsive, and well-led*, do read as a set of conflicting maxims. For example, ‘safe’, ‘effective’, and ‘well-led’ hospitals are often consolidated ones benefitting from economies of scale, but local communities, politicians, and patient associations often resist the closure of what are perceived to be more ‘caring’ and ‘responsive’ district hospitals (Timmins, 2007). In practice, however, the ambiguity of the CQC’s quality goals means that trade-offs between them are rarely acknowledged.

Even when regulators and clinicians can agree on what is ex ante acceptable, the high public saliency and political sensitivity of the NHS mean that there is often little tolerance for adverse outcomes when they come to light. For example, when hospital acquired infections burst onto the headlines in the mid-2000s, the DoH set reduction targets but politicians had little appetite for convincing the public and the media that it would be disproportionately costly to eradicate such infections completely. Despite expert calls to ‘stop pandering to populism about hospital cleanliness’ and to focus instead on the more critical factor of bed occupancy rates (The Lancet, 2007, p. 1102), the Labour Government pledged £50million to ‘deep clean’ every hospital ward in the country (Watt, 2007). Ministerial interventions are so common, explained one senior civil servant, because ‘whenever something blows up, it’s always the government’s problem’. But such interventions tend to undermine previously agreed ex ante compromises between competing quality goals and to reinforce the tendency for new definitions and standards to layer-up on top of each other in unstable configurations that are consequently difficult either to assess or enforce in proportion to risk.

### Assessing care quality ‘risks’

Risk-based regulatory approaches face a second set of problems in assessing healthcare quality. One approach has been to assess quality using peers and inspectors but the objectivity and credibility of these judgements has often been questioned. Traditional forms of professional self-regulation relied on the subjective expert judgement of fellow professionals, but the impartiality of the process was sometimes compromised. For example, the Bristol Royal Infirmary Public Inquiry cited the hospital’s ‘club culture’ as one reason why abnormally high mortality rates among paediatric heart patients went undetected for so long (Kennedy, 2001, para. 8). The rolling quality assurance reviews introduced by CHI in response to that scandal were more formal and independent, but trust managers complained about the calibre and qualifications of CHI’s ad hoc teams of part-time reviewers seconded from elsewhere in the NHS (Day & Klein, 2004).

CHI’s successor, HCC, responded by creating a professional hospital inspectorate that undertook both regular inspections as well as unannounced visits in response to intelligence about risk. This strategy helped counter what one of our informants termed ‘the Queen Mother approach’ of hospitals to ‘rolling out the red carpet’ for the visiting inspectors while ‘sending people who are a bit smelly on leave for the day’. However, when CQC took over responsibility for assessing quality across the health, social care, and mental health sectors, it consolidated the specialist inspectorates for those sectors into a single generalist one. As a result individual inspectors often lacked expertise in the services they were evaluating, not least those 134 inspectors hired without having ‘the core competencies to do the job’ in order to plug chronic staff shortages (Hazell, 2014). In response to damning criticisms (Francis, 2013), the CQC reverted to separate specialist inspectorates for hospitals, primary care, and social care, with hospital inspections now conducted by large multidisciplinary teams of experts.

But healthcare is so complicated that even expert inspectors struggle to detect risks to quality during their necessarily brief inspection visits. As an official involved in healthcare regulation told us, ‘you’d have to be very lucky to spot intermittently bad care if you’re only physically there for 1% of time where that care has been delivered’. For another informant, who had retired after many years in a board-level role in quality regulation, ‘the idea of measuring [quality] through visits, through inspections’ was ‘completely ludicrous’. Detection problems are further compounded by active resistance from health chiefs keen to minimise any local difficulties, as happened at Mid-Staffordshire Trust (Francis, 2013).

A second approach to assessing healthcare quality has been to develop surveillance systems to crunch the wealth of administrative data generated in the NHS and identify statistical anomalies indicating potential risks to quality. The idea, explained one informant involved in their development, was ‘to get away from a model of regulation which waited for disasters to occur and then criticise people’ in favour of ‘a real time understanding of what is happening’ so that ‘you can analyse that something is going wrong before people are actually harmed’. As well as helping regulators ‘make better decisions about when, where, and what to inspect’ (CQC, 2013b, p. 9), another official noted how this statistical surveillance could also ‘provide a component of the evidence for the final judgment’, making quality assessment more objective than relying solely on the subjective judgements of peer reviewers and professional inspectors (cf. Porter, 1995).

Over the last decade, healthcare regulators have developed a series of different statistical surveillance models for identifying risks to quality. The first was developed by the HCC to monitor NHS hospital performance and trigger investigations. Facing government pressure to cut costs and ‘the burden of regulation year on year’ (CQC, 2009, para. 12), the CQC built on that ‘big data’ approach to monitor quality and prioritise inspections across both the health and social care sectors. Its statistical system of ‘quality and risk profiles’ (QRPs) weighted some 1400 qualitative and quantitative indicators to score individual providers for their risk of non-compliance against 16 separate quality standards. After several high-profile detection failures, the CQC (2012, para. 1.2) ‘moved away from an entirely risk-based model’ for targeting unannounced inspections through QRPs and developed a more simplified statistical system to inform what and where to inspect. The new ‘Intelligent Monitoring’ system uses just 150 unweighted indicators to assess risks to quality. However, recent analysis showed that this tool is wrong more often than it is right (Griffiths et al., 2016), and so the CQC (2015e) has announced plans to ‘develop a more comprehensive surveillance model’ to replace Intelligent Monitoring.

There are a number of reasons why statistical surveillance systems have so consistently disappointed (Francis, 2013; House of Commons, Health Committee, 2012; National Audit Office (NAO), 2015a), despite the millions of pounds invested in their development. Part of the problem is that healthcare regulators face ‘formidable difficulties in organising the regulation of the quality of healthcare using the data that are routinely available’ (Bevan & Cornwell, 2006, p. 365). As the CQC (2015a, p. 16) itself recently conceded, ‘existing data … is not yet robust enough across all sectors to be a reliable measure of quality’. For example, while the NHS is awash with data, regulators have relatively little information on private healthcare providers. Data releases typically lag by many months, meaning statistical analyses cannot detect threats to quality in real time. Moreover, most NHS data are compiled at trust level, rather than at the site- and specialty-specific levels where care is actually delivered, so that problems in one part of a trust can be masked by good outcomes elsewhere.

Selecting appropriate quality indicators is also difficult. For example, successive governments have promoted subjective patient experience as a key measure of quality, but medical experts challenge the value of the ‘Friends and Family Test’ and other customer satisfaction surveys (Kmietowicz, 2013). One of our informants was particularly scathing:

The one question that the government insisted we had to ask was ‘were you satisfied with the treatment you received?’ How the **** would the patient know about the treatment they received? …. 250 patients in Manchester were satisfied with Dr. Shipman’s treatment and he killed every one of them. So it’s a wrong measure.

Mortality is more widely accepted as a quality indicator, but constructing robust metrics is fraught with practical challenges such as normalising raw data to take account of patient mix and prior health status (Taylor, 2013). In response to complaints that even normalised mortality rates ‘should not be used to benchmark hospitals’ quality of care’ because they do not consider how many ‘excess deaths’ were preventable (Ramesh, 2015), the health secretary recently announced that the NHS would be creating a new ‘avoidable death’ indicator. However, epidemiologists quickly dismissed that measure as ‘meaningless’ because the sample size is too small to identify problematic trusts or specialties within them (Hazell, 2015). Moreover, the process of statistical benchmarking subtly transforms the very meaning of quality into a question of relative performance. For example, the winsorised z-scoring technique used by HCC and CQC rates the performance of individual units by comparing their performance on a particular indicator against the truncated mean of other units of the same type and assigning a score based on the number of standard deviations from the truncated mean (CQC, 2014a; Spiegelhalter et al., 2012). By this measure, a trust with very long waiting lists would be excellent if waiting lists everywhere else were worse still. ‘Lowest relative risk’ may not be the same as ‘acceptable risk’ when judged against absolute standards of ‘quality care’.

In turn indicators are liable to being ‘gamed’ by regulatees. Thus, the first NHS star rating system distorted hospital performance by encouraging trusts to focus narrowly on the relatively small range of targets that were being measured (Bevan & Hood, 2006). The QRPs responded to this problem by using over 1400 elaborately weighted quantitative and qualitative indicators (CQC, 2012), but this made the QRPs so complicated that they ‘had very little traction with inspectors or really with providers’, an official recalled. On the advice of the consultancy firm McKinsey & Company (DoH, 2015), QRPs were replaced by a new ‘Intelligent Monitoring’ system, which uses a much smaller selection of unweighted indicators. But in this unweighted system, the ‘proportion of Healthcare Workers with direct patient care … vaccinated against seasonal influenza’ bizarrely has as much bearing on hospital risk ratings as the number of ‘never events’ or methicillin-resistant Staphylococcus aureus incidents (CQC, 2014c).

Statistical surveillance faces a final set of challenges in wider public and political intolerance for error in risk assessment. While the most recent House of Commons, Health Committee (2014, p. 6) report claims to ‘accept’ that ‘it will not be possible to pick up every single error or failure’, it also insists that, ‘The surveillance system must identify problems and trigger inspections before they become widely publicised by the media, patient groups or local representatives’ (para 43). However, even egregious instances of poor quality care, such as the infant deaths at Bristol Royal Infirmary or Dr. Shipman murdering his patients, are not necessarily detectable at conventional statistical confidence intervals (Bevan, 2008; Taylor, 2013). In the face of this uncertainty, the CQC could trigger inspections at lower thresholds of probability, but resource limitations mean ‘limits have to be set to guard against too many “false alarms” occurring as a result of random variation’ (CQC, 2014a, p. 9). In its early years, resource pressures forced the CQC to set very high thresholds for triggering inspections (National Audit Office (NAO), 2011), which were subsequently reduced in the wake of the Mid-Staffs scandal. As a former minister explained the CQC was concerned about being ‘held out to dry’ if ‘something goes belly up’. But this concern with ‘safeguard[ing] their own position’, as that informant termed it, sits uneasily with risk-based rationales and comes with high opportunity costs.

### Enforcing quality

Risk-based regulatory approaches face a third set of difficulties in calibrating regulatory enforcement in terms of risk. Since the CQC recognises that ‘it is not feasible or proportionate to follow up every single breach of standards’ (CQC, 2015b, p. 5), it uses a formal risk matrix that assesses the probability and consequences of regulatory breaches to ‘select the appropriate enforcement power’. These powers currently range from simple advice or public disclosure to more punitive civil enforcement measures, such as warnings and requirement notices, and *in extremis* fines, criminal prosecutions, and cancellation of registration (CQC, 2015c). This enforcement pyramid of escalating sanctions can then, in theory, be matched to the competence, capacity, and willingness of regulatees to meet regulatory requirements (cf. Ayres & Braithwaite, 1992).

In practice, however, this risk-based model has proved hard to implement. One reason is that risk-based enforcement can fall foul of equalities requirements. For example, while it is not unknown for disproportionately high levels of abuse complaints to be made against male healthcare professionals, the Professional Standards Authority for Health and Social Care (PSA, 2015, p. 12) warns that, ‘Taking regulatory action based on an apparent statistical correlation between harmful behaviour and a group defined by, say its age or ethnicity, is likely to be discriminatory’.

Even when risk-based enforcement is permissible, regulators have hesitated to deploy enforcement sanctions. As a senior civil servant from the DoH observed to us:

After the Mid-Staffordshire disaster, there was a view that mechanisms for monitoring risks in terms of quality were not good. … Actually the main problem was addressing the failures …. There had been previous reports, including from the Healthcare Commission that had identified those problems, but nobody really reacted.

Regulators have hesitated to sanction partly for fear of undermining the continuity of healthcare provision. Financial penalties can be self-defeating if they exacerbate under-resourcing, while the ultimate sanction of closure is an unrealistic threat for hospital, ambulance, and mental health trusts who are often local monopoly providers. However, even closing GP surgeries, which are more numerous, is often politically controversial (Liverpool Echo, 2015). The 2012 Health and Social Care Act addressed that problem by giving the CQC power to put trusts into ‘special measures’, which can include imposing new management and forced mergers with more successful trusts. Within 2 years, some 21 NHS hospital trusts, or more than 10% of all acute trusts in England, had been put into special measures. But with only a limited number of nearby trusts available to support those in special measures, there are clearly limits to this last resort approach.

More compliance-oriented enforcement tools for incentivising behaviour change in the NHS have not been particularly effective either. One reason is that until recently, the CQC was unable to offer much education and advice because its generalist inspectorate lacked the expertise to do so. Moreover, even improvement notices were liable to be ignored, because, as a former special advisor explained to us, inspectors ‘had no credibility with the doctors’. This might have changed in 2013 when the CQC’s inspectorate was reformed with a dedicated expert hospital inspectorate. However, it is not clear that ignorance of what to do is the major cause of poor quality care in NHS hospitals. Hospitals are not like restaurants owned by uninformed regulatees who can benefit from the advice of expert food safety inspectors on meeting food hygiene standards (Yapp & Fairman, 2006). There is no shortage of technical expertise in hospitals; rather it is their size, complexity, and multiple goals that make it difficult to ensure consistent quality across the wide range of services they provide.

Likewise, naming and shaming mechanisms, such as publishing quality ratings, have created little demand-side pressure to drive up quality standards (Laverty et al., 2012). The star ratings published by the CHI and HCC from 2001 to 2005, like the Annual Health Check that succeeded them, were easy to understand, but they rated entire trusts and, therefore, provided little help to patients selecting the best hospital for hip replacements. Even advocates of patient choice concede that point. As one former health minister put it, ‘The idea that my hospital is either safe or not safe is just not real; health and social care is so big, so diverse’.

Having been ordered to abandon the HCC’s Annual Health Check because of the limited value of single-word summary judgments of hospital quality (West, 2010), the CQC published inspection findings for its 16 different quality standards. However, the public struggled to make sense of the sheer number of standards (Nuffield Trust, 2013). Under pressure from the government to return to a simpler Ofsted-style summary judgement (DoH, 2012), the CQC now publishes consolidated inspection grades and risk ratings, but, as before, that is unlikely to help patients select the best provider.

Ratings have also faced fierce sectorial resistance from medical professionals. Describing the construction of CHI’s first star rating system, a former DoH advisor recalled in his interview with us that:

When we started this, we … thought we were just adding another piece of bureaucracy, but … inspecting one of the best heart surgeons in the world is different from inspecting a primary school teacher. There was a lot of … anger [and] noise in the system. Instead of recognising the interest of CHI, people were rather ‘how dare you?’

One notable example was in 2015, when GPs complained fiercely about the accuracy and effects on public confidence of the CQC’s published statistical risk ratings of surgeries (Millett, 2015). The CQC was subsequently forced to publish only raw statistics and to ‘change the language used to highlight variation between practices so that it does not imply a risk to patient safety’ (CQC, 2015d). But just as the Labour Prime Minister Callaghan in 1976 famously questioned whose interests the ‘secret garden’ of educational professionals was serving, so doctors have found it increasingly hard to close off their own private Eden. For example, the Society for Cardiac Surgery began publishing its own risk-adjusted mortality rates for cardiac surgeons in 2007 in an effort to pre-empt the sort of externally imposed rating schemes that NHS England began publishing for other surgical specialisms in 2013.

Publicly naming and shaming NHS services is also politically sensitive. As former CQC chair Baroness Young testified to the Francis Inquiry:

regulating services provided by a Government Minister, was … always going to be incredibly fraught, because inevitably both the Department and Ministers were torn between wanting good, strong independent regulation of healthcare and knowing that … from time to time they would be put in the dock and found wanting. (Francis, 2013, p. 941)

Another current regulator described to us the difficult conversations with ministers about what to do:

in the run up to the election … the Prime Minister and secretary of state are desperate to keep the health sector quiet … so we do get a fair amount of ‘you’re not going to do anything that’s going to be noisy, are you?’ to which we tend to go back with … ‘if we don’t do something in this particular case, it’s likely that patients are dying, you don’t really want that on your conscience, do you?’

Given these difficulties, the risk-based enforcement pyramid provides little help to regulators struggling to ensure care quality in the NHS.

## Discussion

For many years now, a central dogma of international regulatory reform programmes has been that risk-based regulation is ‘better regulation’ and that it will help regulators define regulatory goals, monitor performance, and secure compliance with them in more effective, economical, proportionate, and publicly legitimate ways. However our findings show that risk-based reforms of quality regulation in the NHS have consistently failed to deliver on those promises.

There are at least four reasons why risk-based regulation has not worked. First, ambiguities in the very idea of healthcare quality have made it impossible to define clear and enduring goals for regulating it in terms of risk. The instability of risk-based regulatory goals is partly due to healthcare being a matter of universal concern, involving powerful interest groups and, in the case of the NHS, assuming totemic significance as a symbol of national identity. However, the tendency for competing regulatory desiderata to get layered on top of each other in unstable configurations owes more to the difficulties so influentially described by Donabedian (1988) in defining ‘quality’ than to the political dynamics of healthcare per se. After all, quality regulation in other domains, such as higher education, has suffered from similar difficulties in defining clear and uncontested goals in terms of competing input, process, and outcome measures.

Second, regulators have struggled to agree on, or sometimes even acknowledge, the trade-offs between competing desiderata implicit in the concept of ‘acceptable risk’. While such trade-offs are accepted in some policy domains such as workplace safety, it has proved much harder to secure agreement in healthcare, not least because of the difficulty in reconciling conflicts between public expectations of universal provision of safe healthcare, professional views of clinical effectiveness, and managerial concerns for cost control. Moreover, even if ex ante agreement could be reached on what risks are in principle acceptable, the high public saliency and political sensitivity of the NHS means that adverse outcomes are rarely regarded as acceptable, ex post, when they come to light.

Third, despite access to what is probably the most comprehensive database on outcomes and performance for any regulated sector in the world, NHS regulators have also struggled to assess risks to quality, identify which providers are at greatest risk of failing to meet quality standards, or prioritise inspections accordingly. Reasons include difficulties in: making credible inspection judgements about complex healthcare organisations; devising appropriate indicators to capture outcomes for patients and ‘what matters’ to them; interpreting vast quantities of heterogeneous and conflicting data; and adapting measurement methods to combat gaming and frequent changes in policy and in the organisation of healthcare and regulation.

Fourth, calibrating regulatory interventions according to risk has also proved impractical. Punitive responses to non-compliance can compromise the quality and continuity of healthcare delivery, making any intervention – proportionate to risk or otherwise – difficult. More compliance-oriented levers for improving quality such as earned autonomy, education and advice, or public disclosure, however, can create perverse incentives, alienate expert clinical staff or fail to supply sufficiently granular information about performance to be useful in leveraging change in a sector characteristically dominated by monopolistic provision. Those problems have been compounded by the scale of the problems in some healthcare sectors. For example, with more than 80% of the acute trusts inspected in England over the last 2 years found to be ‘inadequate’ or to ‘require improvement’ (Griffiths et al., 2016), the regulatory challenge is not one of targeting the odd ‘bad apple’ but fixing the whole ‘barrel’.

Why then, after a decade of repeated failures, are healthcare regulators still committed to risk-based regulation? There are perhaps three answers to that question, which draw inspiration from neo-institutionalist theory and its emphasis on coercive, normative, and mimetic drivers of organisational behaviour (DiMaggio & Powell, 1983). First, the CQC has no choice. Keen to cut costs and reduce regulatory burdens, successive governments in the UK have aggressively promoted risk-based reforms. Indeed, for nearly a decade now, all UK regulators have been legally mandated to ‘base their regulatory activities on risk’ (BIS, 2014, para. 3). While departing from this ‘best practice’ is not an option, ambiguity about what it actually means has allowed care quality regulators to maintain their commitment to being ‘risk-based’ despite remarkable instability in their organisational structure, regulatory models, and policy tools.

A second reason for the continued commitment to risk-based regulation is its normative appeal. Like new toothpaste brands, risk-based tools invariably promise to be cheaper, better targeted, and more effective than their predecessors. Faced with the Sisyphean task of ensuring quality across a hugely complex, crisis-prone, and politically sensitive sector, healthcare regulators find those promises particularly alluring. At the same time ceaseless reform, both of the NHS and its regulators, has eroded institutional memory and helped to shield risk-based regulation from critical tests of its core promises. Thus, proponents of statistical surveillance still ‘find it hard to believe that there are not some prior indications of when quality of care may be at risk’ (Bardsley, 2016, p. 2), even in the face of repeated failures and clear evidence that ‘big data’ analytics has not worked (Griffiths et al., 2016).

Third, the continuing recourse to risk-based approaches also reflects mimetic pressures to secure legitimacy through conformity with prevailing organisational fashions. Rather than leading to questions about the appropriateness of this strategy, successive failures of risk-based quality regulation have instead led to changes in institutional structure and leadership. Organisations in that context are classically vulnerable to the mimetic appeal of technical systems peddled by international management consultancies (Larner & Laurie, 2010). Certainly, the CQC often brags about its ‘leading-edge thinking’ (Public Accounts Committee (PAC), 2015) on statistical surveillance, even though its Intelligent Monitoring system – which it procured from the consultancy firm McKinsey through a faulty tendering process (DoH, 2015) – does not actually work. As Porter (1995) has observed, it is not uncommon for organisations to look to numerical and calculative rationales to augment their legitimacy irrespective of methodological validity.

But more than simply wowing them with numbers, risk-based approaches are also appealing to healthcare regulators as a way of accounting for the inevitable limits of what they can do to ensure quality (Rothstein, 2006). After all the language of risk is a powerful way of deflecting blame by rationalising adverse events as outcomes that were in principle acceptable ex ante, given limited resources and competing demands upon them. The problem is that in healthcare, despite lip service sometimes paid to those limits, expectations are high, the need for trade-offs often denied, and adverse quality outcomes often treated as regulatory failures. In this context of restricted tolerability of risk, risk-based approaches to regulating the NHS are likely to fail.

## Conclusion

In this article, we have used the case of regulating health care quality in the NHS to identify some more general challenges facing risk-based approaches to healthcare regulation. From our case, it is possible to distil several preconditions for successful risk-based regulation:

- Regulatory goals must be clear and trade-offs between them amenable to ex ante agreement;
- Regulators must be able to reliably assess the probability and consequences of adverse outcomes that are potentially unacceptable;
- Regulators must have a range of enforcement tools that they can deploy effectively in response to increasing risk;
- There must be political tolerance ex post for adverse outcomes that had been defined ex ante as acceptable risks.

Many of these preconditions do not hold for healthcare regulation in England. The symbolic importance of the NHS creates difficulties for ministers in both publicly acknowledging the limits to what the NHS can deliver (i) and in avoiding blame when things go wrong (iv). Accepting limits and tolerating failures might be less of an issue in other healthcare systems where accountabilities are more diffuse, such as in the US and Germany. However, those systems are more fragmented, which creates other barriers to risk-based regulation by making comprehensive risk assessment (ii) and risk-based enforcement (iii) more difficult.

The lessons from this research go well beyond healthcare. Risk promises to make regulation more effective and proportionate across policy domains. However, risk-based reforms are unlikely to succeed when goals are ambiguous or contested, failures hard to spot in advance, and political tolerance of adverse outcomes uncertain.
